# The association of atherosclerotic cardiovascular disease and statin use with inflammation and treatment outcomes in tuberculosis

**DOI:** 10.1038/s41598-021-94590-x

**Published:** 2021-07-27

**Authors:** Vignesh Chidambaram, Jennie Ruelas Castillo, Amudha Kumar, Justin Wei, Siqing Wang, Marie Gilbert Majella, Akshay Gupte, Jann-Yuan Wang, Petros C. Karakousis

**Affiliations:** 1 Division of Infectious Diseases, Department of Medicine, Johns Hopkins School of Medicine, Koch Cancer Research Building, 1550 Orleans St., Room 110, Baltimore, MD 21287 USA; 2 Department of Internal Medicine, University of Arkansas for Medical Sciences, Little Rock, AR USA; 3 Department of Preventive and Social Medicine, Jawaharlal Institute of Postgraduate Medical Education and Research, Pondicherry, India; 4 Department of Internal Medicine, National Taiwan University Hospital, Taipei, Taiwan; 5 Department of International Health, Johns Hopkins Bloomberg School of Public Health, Baltimore, MD USA

**Keywords:** Tuberculosis, Atherosclerosis, Cerebrovascular disorders, Coronary artery disease and stable angina

## Abstract

Tuberculosis (TB) and atherosclerotic cardiovascular disease (ASCVD) have a close epidemiological and pathogenetic overlap. Thus, it becomes essential to understand the relationship between ASCVD and TB outcomes. From our retrospective cohort on drug-susceptible TB patients at the National Taiwan University Hospital, we assessed the association of pre-existing ASCVD (coronary artery disease (CAD) and atherothrombotic stroke (ATS)) with 9-month all-cause and infection-related mortality and the extent of mediation by systemic inflammatory markers. We determined the effect of pre-existing ASCVD on 2-month sputum microbiological status. Among ASCVD patients, we assessed the association of statin use on mortality. Nine-month all-cause mortality was higher in CAD patients with prior acute myocardial infarction (CAD^+^AMI^+^) (adjusted HR 2.01, 95%CI 1.38–3.00) and ATS patients (aHR 2.79, 95%CI 1.92–4.07) and similarly, for infection-related mortality was higher in CAD^+^AMI^+^ (aHR 1.95, 95%CI 1.17–3.24) and ATS (aHR 2.04, 95%CI 1.19–3.46) after adjusting for confounding factors. Pre-existing CAD (AMI^-^ or AMI^+^) or ATS did not change sputum culture conversion or sputum smear AFB positivity at 2 months. The CAD^+^AMI^+^ group had significantly higher levels of CRP at TB diagnosis in the multivariable linear regression analysis (Adjusted B(SE) 1.24(0.62)). CRP mediated 66% (*P* = 0.048) and 25% (*P* = 0.033) of the association all-cause mortality with CAD^+^AMI^−^ and CAD^+^AMI^+^, respectively. In summary, patients with ASCVD have higher hazards of 9-month all-cause and infection-related mortality, with elevated serum inflammation mediating one to three-quarters of this association when adjusted for confounders. Statin use was associated with lower all-cause mortality among patients with ASCVD.

## Introduction

Tuberculosis (TB) is a global disease, with low- and middle-income countries contributing nearly 98% of incident cases^1^. In parallel, these regions account for almost 80% of global cardiovascular mortality^2,3^. Patients with TB, either active TB disease or latent TB infection, have an increased risk of developing atherosclerotic cardiovascular diseases (ASCVD), such as coronary artery disease (CAD)^4–6^, including acute myocardial infarction (AMI)^7,8^, and acute ischemic stroke^9,10^, as well as mortality due to these diseases^11^. However, the effect of pre-existing ASCVD on TB treatment outcomes is uncertain.

Elevated systemic inflammatory markers, such as C-reactive protein (CRP), total white blood cell count (WBC), and neutrophil–lymphocyte ratio (NL ratio), are elevated and predict poor outcomes in ASCVD patients^12–16^. CRP and other inflammatory markers also correlate with sputum mycobacterial load^17^, and portend a poor prognosis^18^ and increased mortality^19–22^ in TB patients. Thus, there is a need to assess the potential role of systemic inflammation in the association between pre-existing ASCVD and TB treatment outcomes.

Statins, apart from the role in primary and secondary CAD prevention, are known to reduce systemic inflammation^23^. Preclinical models have shown that statins enhance autophagy and phagosome maturation in *Mycobacterium tuberculosis*-infected macrophages and reduce the bacillary burden in human macrophages. As adjunctive therapy, statins improve sterilizing activities of the first-line anti-tubercular regimen in murine models^24–27^. Though systematic reviews have shown that statin use reduces the incidence of active TB ^28,29^, there is no clinical evidence on whether statins have salutary effects in TB patients after adjusting for confounding factors^30^.

In our study, we assessed the association of pre-existing ASCVD with all-cause and infection-related mortality during the first nine months of TB treatment and evaluated to what extent the levels of systemic inflammatory markers mediate this association. We also determined the effect of pre-existing ASCVD on 2-month sputum culture and acid-fast bacilli (AFB) smear conversion. Among patients with pre-existing ASCVD, we assessed the association of statin use with 9-month mortality.

## Methods

### Study design and population

Our retrospective cohort included all consecutive adults (aged > 18 years) with culture-confirmed drug-susceptible TB, treated according to American Thoracic Society guidelines^31^ at the National Taiwan University Hospital (NTUH), a referral center in Taipei city from 2000 to 2016, with no specific exclusion criteria^32^. All data were obtained from the NTUH database. The study was approved by the institutional review boards (IRB) at Johns Hopkins University and NTUH and the study methods were carried out in accordance with the IRB guidelines and regulations. As this was a retrospective chart review, informed consent from the study subjects was waived off by the IRBs at NTUH and Johns Hopkins School of Medicine.

### Baseline characteristics

Data were obtained on baseline characteristics, such as age, sex, body mass index (BMI), diabetes mellitus (DM), hypertension (HTN), cancer, chronic kidney disease (CKD), chronic obstructive pulmonary disease (COPD), asthma, smoking, alcohol use disorder, solid organ or bone marrow transplantation, HIV status, baseline sputum smear for AFB (0 to 4 +), cavitary disease on chest radiography, and prior TB history.

### Exposures

The three exposures assessed in our study are as follows: (1) CAD: the diagnosis of pre-existing CAD was based on either angiographic evidence of CAD, admission ICD codes, or review of outpatient medical records. For this exposure, the patients were stratified into three groups: No evidence of CAD (CAD-), CAD without history of AMI (CAD^+^AMI^−^) and CAD with history of AMI (CAD^+^AMI^+^); (2) Atherothrombotic stroke (ATS): The ATS subtype of stroke, based on admission ICD codes or outpatient medical records, was our exposure of interest; (3) Statin use among patients with ASCVD (CAD or ATS): For this study, we defined pre-existing ASCVD as the presence of pre-existing CAD or ATS. Exposure for the “intention-to-treat” analysis for statin use was defined as the use of any of the following equivalent doses of moderate-intensity statin therapy^33^: atorvastatin 10 mg, simvastatin 20 mg, pravastatin 40 mg, rosuvastatin 10 mg, lovastatin 40 mg, fluvastatin 80 mg or pitavastatin 2 mg^33^ for a minimum of 2 weeks (14 doses) in the first month of TB treatment. Exposure for the “per-protocol” analysis was defined as the use of any of the above doses of statins for > 80% (7.2 months) of the 9 months following the initiation of TB treatment, or > 80% of the duration of follow-up if lost to follow-up or death occurred before nine months.

### Systemic inflammatory markers

C-reactive protein (CRP)(mg/dL), total leukocyte count (WBC) (× 10^3^/µL) and neutrophil–lymphocyte ratio (NL ratio) available at baseline were documented. Any test result for the inflammatory markers within 30 days of TB diagnosis was considered as baseline.

### Outcomes

Primary outcomes were all-cause and infection-related mortality during the first 9 months of TB treatment. Infection-related mortality was a composite outcome of death due to pneumonia, sepsis, or TB. Secondary outcomes included sputum AFB smear positivity and culture positivity at 2 months after treatment initiation.

### Statistical analysis

Patient characteristics, stratified into three groups of CAD status (non-CAD, CAD^+^AMI^−^, CAD^+^AMI^+^), were compared using ANOVA for normally distributed data, Kruskal–Wallis test for non-normally distributed data, and chi-square (χ^2^) test for categorical variables. Patient characteristics stratified by the presence of ATS, the second exposure of interest, were compared using a two-sided t-test and χ^2^ test for continuous and categorical variables, respectively. Kaplan–Meier analysis and Cox proportional hazards regression was used to measure the association between the above exposures and all-cause and infection-related mortality in separate models. Detailed statistical methods are described in the supplementary document (section I). The association between the above exposures and sputum-smear and culture positivity at 2 months were analyzed using univariable and multivariable logistic regression. Potential confounders for multivariable analyses were identified by literature review and by exploratory univariable data analysis at *P* < 0.05 significance, depending on the assessed exposure.

The association of pre-existing CAD and ATS, and serum inflammatory markers, namely CRP, WBC, and NL ratio, was analyzed using univariable and multivariable linear regression analyses. We constructed a causal directed acyclic graph (cDAG) to represent our proposed mediation hypothesis linking the exposure (pre-existing CAD^+^AMI^−^ or CAD^+^AMI^+^ or ATS) and the outcome (all-cause and infection-related mortality during TB treatment) by using inflammatory markers (measured by CRP, NL ratio, WBC count) as the potential mediators. The confounders identified above were added to the model. We estimated the path coefficients using structural equation modelling (SEM). The effect on ASCVD on mortality that is mediated through the inflammatory markers was considered the “indirect effect,” and that mediated by all other factors constituted the “direct effect”. The statistical significance of the indirect effects was assessed by the post-estimation “medsem” command^34^ in STATA, which utilizes the Zhao, Lynch & Chen's approach^35^ to testing mediation through the Monte-Carlo simulations (5000 reps).

Among patients with pre-existing ASCVD, we measured the association of statin use, classified according to the intention-to-treat and per-protocol definitions, and all-cause mortality and infection-related mortality using separate univariable and multivariable Cox regression models. In order to appropriately assign statin exposure, only patients who survived beyond the first-month post-TB treatment initiation were included. Sensitivity analyses were conducted by: (i) including patients who died within the first month of TB treatment; and (ii) excluding patients who did not receive the required dose of statin from the comparison group. Statistical analyses were performed using STATA/IC 16.0 software (StataCorp, College Station, Texas).

## Results

### Patient characteristics and outcomes

**Figure 1:**
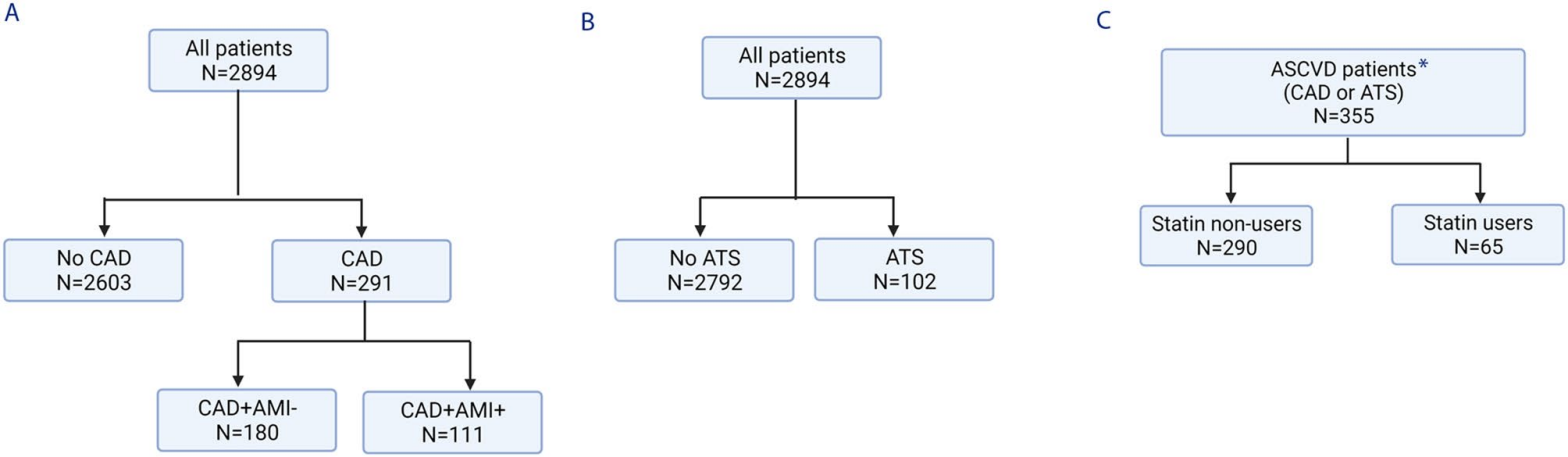
Flow diagram for patient stratification. (**A**) Stratification based on the diagnosis of pre-existing coronary artery disease (CAD). (**B**) Stratification based on the diagnosis of pre-existing atherothrombotic stroke (ATS). (**C**) Stratification based on statin use among patients with Atherosclerotic cardiovascular disease (ASCVD). CAD^+^AMI^−^, Pre-existing coronary artery disease without prior acute myocardial infarction; CAD^+^AMI^+^, Pre-existing coronary artery disease with prior acute myocardial infarction. ^*^38 patients had both pre-existing CAD and ATS.

Among 2,894 culture-confirmed, drug-sensitive pulmonary TB cases, with a median (IQR) age of 66.6 (49.1–77.8) years, 355 patients had pre-existing ASCVD (Fig. 1). Among the 355 patients, 291 patients had CAD, while 102 patients had a history of ATS. Thirty-eight patients had a history of both CAD and ATS. Among 291 patients with CAD, 180 patients (6.2%) had no prior AMI (CAD^+^AMI^−^ group), and 111 patients (3.8%) had prior AMI (CAD^+^AMI^+^ group) (Table 1 and Fig. 1).

**Table 1:** Patient characteristics stratified by coronary artery disease (CAD) and atherothrombotic stroke (ATS).

Study characteristics	Measure	All patients (N = 2894)	Coronary Artery Disease (CAD)				Atherothrombotic stroke (ATS)		
			Non-CAD (n = 2603)	CAD^+^ AMI^-^(n = 180)	CAD^+^ AMI^+^ (n = 111)	*P*-value	Non-ATS (n = 2792)	ATS (n = 102)	*P*-value
Age (years)	Median (IQR)	66.6 (49.1–77.8)	64.3 (47.3–76.9)	78.1 (72.2–84.4)	71.4 (58.7–82.8)	< 0.001	65.9 (48.4–77.5)	77.8(71.4–86.3)	< 0.001
Male Sex	No (%)	1975 (68.2%)	1756 (67.5%)	140 (77.8%)	79 (71.2%)	0.013	1910 (68.4%)	65 (63.7%)	0.318
BMI	Mean (SD)	21.1 (4.2)	21.2 (4.2)	21.8 (3.8)	21.8 (3.5)	0.019	21.3 (2.9)	21.5 (4.4)	0.545
Smoking	No. (%)	930 (40.5%)	822(40.3%)	73(44.5%)	35(38.5%)	0.523	897 (40.6%)	33 (38.4%)	0.681
Alcoholism	No. (%)	81 (2.8%)	74 (2.8%)	4 (2.2%)	3 (2.7%)	0.886	78 (2.8%)	3 (3%)	0.901
DM	No. (%)	533 (18.5%)	434 (16.7%)	72 (40%)	27 (24.3%)	< 0.001	489 (17.5%)	44 (44%)	< 0.001
HTN	No. (%)	1052(36.4%)	842 (32.4%)	145 (80.6%)	65 (58.6%)	< 0.001	968 (34.7%)	84 (84%)	< 0.001
Cancer	No. (%)	459 (15.9%)	398 (15.3%)	38 (21.1%)	23 (20.7%)	0.044	439 (15.8%)	20 (20%)	0.253
CKD Stage ≥ 3	No. (%)	970 (33.5%)	858 (32.9%)	77 (42.8%)	35 (31.5%)	0.024	922 (33%)	48 (48%)	0.002
Asthma	No. (%)	123 (4.2%)	99 (3.8%)	15 (8.3%)	4 (3.6%)	0.012	114 (4.1%)	4 (4%)	0.968
COPD	No. (%)	451 (15.6%)	365 (14.0%)	66 (36.7%)	20 (18.0%)	< 0.001	428 (15.3%)	23 (23%)	0.037
Bronchiectasis	No. (%)	121 (4.2%)	106 (4.1%)	9 (5.0%)	6 (5.4%)	0.672	119 (4.3%)	2 (2%)	0.267
Pneumoconiosis	No. (%)	35 (1.2%)	30 (1.2%)	4 (2.2%)	1 (0.9%)	0.426	35 (1.3%)	0 (0%)	0.260
Liver cirrhosis	No. (%)	50 (1.7%)	45 (1.7%)	2 (1.1%)	3 (2.7%)	0.599	49 (1.8%)	1 (1%)	0.570
History of transplant	No. (%)	26 (.9%)	20 (0.8%)	3 (1.7%)	3 (2.7%)	0.057	25 (0.9%)	1 (1%)	0.913
HIV	No. (%)	65 (2.3%)	61 (2.3%)	1 (0.6%)	3 (2.7%)	0.278	65 (2.3%)	0 (0%)	0.123
CAD	No. (%)	291 (10.0%)	0 (0%)	180(100%)	111(100%)	-	253 (9.1%)	38 (37.3%)	< 0.001
ATS	No. (%)	102 (3.5%)	64 (2.5%)	21(11.7%)	17(15.3%)	< 0.001	0 (0%)	102 (100%)	-
**Baseline lipid levels**									
LDL (n = 170)*	Mean (SD)	90.7 (36.9)	91.5 (37.9)	89.3 (39.3)	84.5 (21.9)	0.792	91.2 (37.6)	83.3 (23.3)	0.492
HDL (n = 249)*	Mean (SD)	35.5 (14.8)	35.1 (14.3)	38.9 (14.2)	38.3 (21.3)	0.528	35.5 (14.3)	36.3 (21.7)	0.837
TCHOL (n = 429)*	Mean (SD)	152.2 (51.3)	152.4 (53.0)	155.3 (42.6)	144.2 (28.1)	0.623	152.5 (52.0)	145.7 (33.0)	0.057
TG (n = 495)*	Mean (SD)	109.3 (79.1)	110.6 (81.1)	95.4 (49.7)	111.1 (85.6)	0.928	110.9 (80.4)	81.7 (44.3)	0.067
**TB disease characteristics**									
Initial AFB smear positivity	No. (%)	1215 (42.8%)	1096 (42.8%)	62 (35.2%)	57 (53.8%)	0.009	1179 (42.9%)	36 (37.9%)	0.330
Initial AFB smear grade	Median (IQR)	0 (0–2)	0 (0–2)	0 (0–1)	1 (0–3)	0.002	0 (0–2)	0 (0–1)	0.127
Prior TB	No. (%)	98 (6.1%)	83 (5.8%)	10 (9.6%)	5 (6.8%)	0.291	96 (6.3%)	2 (3.2%)	0.318
Cavitary disease	No. (%)	413 (14.3%)	390 (14.9%)	13 (7.2%)	10 (9.0%)	0.004	406 (14.5%)	7 (7%)	0.034
**Cardiovascular drug use**									
Metformin users	No. (%)	252 (8.7%)	208 (7.9%)	33(18.3%)	11 (9.9%)	< 0.001	236 (8.5%)	16 (16%)	0.008
Statin users	No. (%)	154 (5.3%)	97 (3.7%)	39 (21.7%)	18 (16.2%)	< 0.001	140 (5.0%)	14 (14%)	< 0.001
Calcium channel blocker users	No. (%)	391 (13.5%)	308(11.8%)	52(28.9%)	31(27.9%)	< 0.001	351 (12.6%)	40 (40%)	< 0.001
**Outcomes**									
All-cause mortality	No. (%)	544/2667 (20.4%)	455 (19.0%)	50 (29.6%)	39 (37.1%)	< 0.001	499 (19.4%)	45 (44.1%)	< 0.001
Infection-related mortality	No. (%)	303/2667 (11.4%)	252 (10.5%)	28 (16.6%)	23 (21.9%)	< 0.001	279 (10.9%)	24 (25.3%)	< 0.001
2-month Sputum-culture positivity	No. (%)	265/1640 (16.2%)	240 (16.2%)	11 (10.8%)	14 (23.3%)	0.107	257 (16.2%)	8 (14.8%)	0.785
2-month Sputum-smear AFB positive	No. (%)	118/1640 (7.2%)	109 (7.4%)	4 (3.9%)	5 (8.3%)	0.402	114 (7.2%)	4 (7.4%)	0.951

AFB: Acid-fast bacilli; BMI: Body mass index; ATS: Atherothrombotic stroke, CAD: Coronary artery disease; CAD + AMI + : Coronary artery disease with history of Acute myocardial infarction; CAD + AMI-: Coronary artery disease without history of Acute myocardial infarction; CCB: Calcium channel blocker; CCI: Charlson comorbidity index; CHF: Congestive heart failure; CKD: Chronic kidney disease; COPD: Chronic obstructive pulmonary disease; CVA: Cerebrovascular accident; DM: Diabetes mellitus; HDL: High density lipoprotein cholesterol; HIV: Human immunodeficiency virus; HTN: Hypertension; IQR, Interquartile range; LDL: Low density lipoprotein cholesterol; PVD: Peripheral vascular disease; SD: Standard deviation. * Lipid levels were available only for a subset of the population.

During the first 9 months after TB treatment initiation, a total of 544/2667 patients (20.4%) died of all causes, and 303/2667 patients (11.4%) died of infection-related causes, comprising 55.7% (303/544 patients) of all deaths. Among this group, 43 patients (14.2%) died due to TB-related causes, 124 patients (40.9%) due to pneumonia, and 136 patients (44.9%) due to sepsis. At 2 months, 265/1640 (16.2%) patients had positive culture results, and 118/1640 (7.2%) patients had positive sputum smear AFB.

### Stratification based on pre-existing CAD and AMI

#### Patient characteristics

Compared to patients in the non-CAD group, patients in the CAD^+^AMI^+^ and CAD^+^AMI^−^ groups were older (median age; 64.3 vs. 78.1 vs. 71.4 years, respectively; *P* < 0.001), more likely to be male (67.5% vs. 77.8% vs. 71.2%, respectively; *P* = 0.013) and had a higher BMI (mean BMI (kg/m^2^); 21.2 vs. 21.8 vs. 21.8, respectively; *P* = 0.013). Comorbidities, such as DM, HTN, cancer, CKD, asthma, and COPD, were more prevalent in the CAD groups. Sputum smear AFB positivity at baseline was highest in the CAD^+^AMI^+^ group (53.8%) (Table 1). The non-CAD group had a higher proportion of patients with cavitary TB disease at baseline compared to the CAD^+^AMI^−^ and CAD^+^AMI^+^ groups (14.9% vs. 7.2% vs. 9.0%, respectively; *P* < 0.001) (Table 1).

#### Mortality

**Figure 2:**
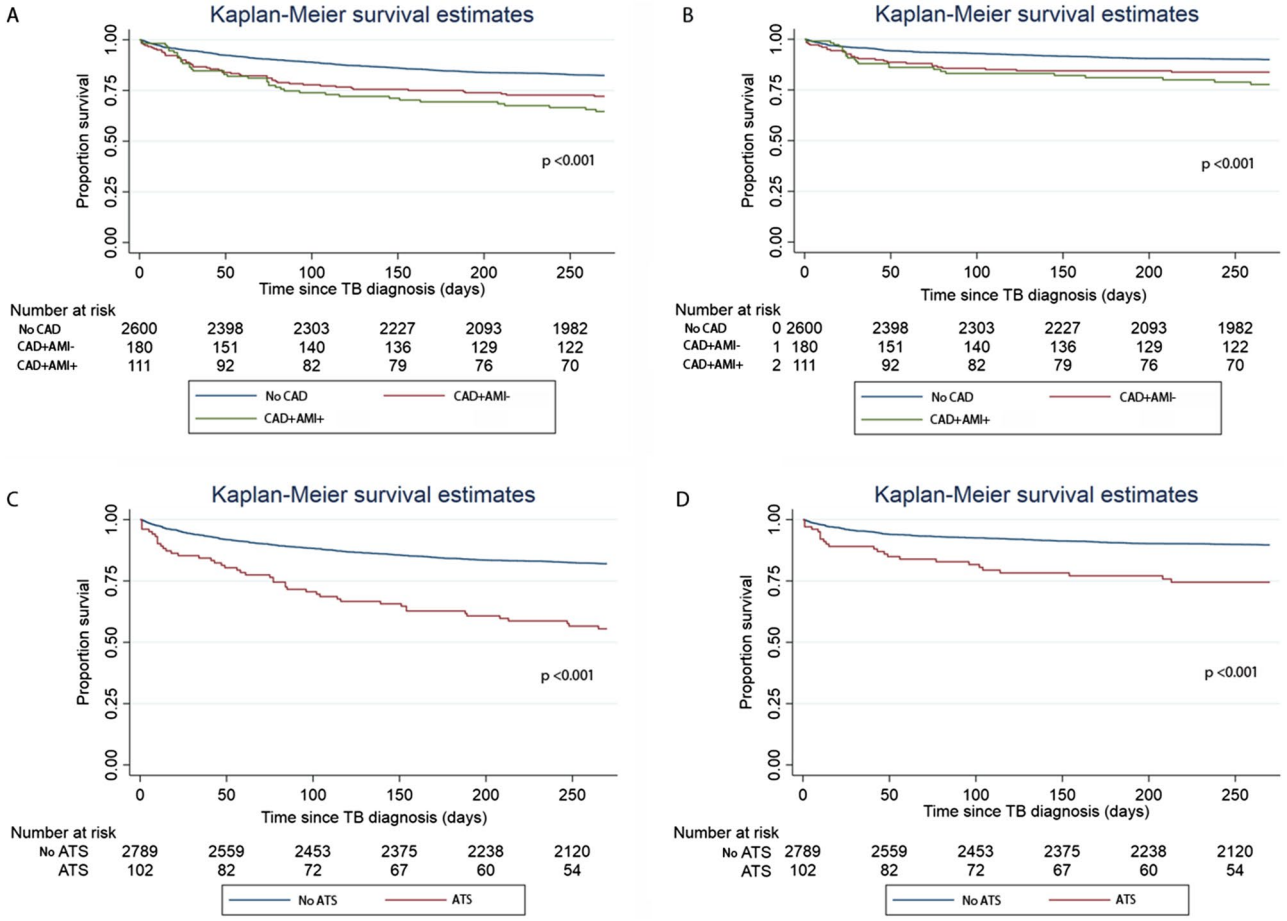
Kaplan Meier analysis on the association of pre-existing coronary artery disease (CAD) with (**A**) all-cause mortality and (**B**) infection-related mortality. Association of pre-existing atherothrombotic stroke (ATS) with (**C**) all-cause mortality and (**D**) infection-related mortality.CAD^+^AMI^−^, Pre-existing coronary artery disease without prior acute myocardial infarction; CAD^+^AMI^+^, Pre-existing coronary artery disease with prior acute myocardial infarction.

There was a higher all-cause mortality in the CAD^+^AMI^+^ group (39/105 patients, 37.1%) and CAD^+^AMI^−^ group (50/169 patients, 29.6%) compared to the non-CAD group (455/2393 patients, 19.0%) (*P* < 0.001) by χ^2^ test (Table 1). The log-rank test of the Kaplan–Meier analysis showed that patients in the CAD^+^AMI^−^ and CAD^+^AMI^+^ groups had significantly shorter survival compared to patients without CAD (Fig. 2A; *P* < 0.001). After adjusting for age, gender, BMI, DM, HTN, cancer, CKD, asthma, COPD, liver cirrhosis, transplant status, sputum AFB status and cavitary disease at baseline, metformin use, statin use, calcium channel blocker use, patients in the CAD^+^AMI^−^ group had a HR of 1.31 (95%CI 0.91–1.89) and the CAD^+^AMI^+^ group had a HR of 2.04 (95%CI 1.38–3.00) for all-cause mortality (Table 2). Patients with CAD^+^AMI^−^ and CAD^+^AMI^+^ had earlier infection-related mortality by the log-rank test of the Kaplan–Meier analysis (Fig. 2B; *P* < 0.001). After adjusting for confounders, the CAD^+^AMI^−^ group had a HR of 1.36 (95%CI, 0.84–2.21) and the CAD^+^AMI^+^ group had a HR of 1.95 (95%CI, 1.17–3.24) for infection-related mortality compared to the non-CAD group (Table 2).

**Table 2:** Association of CAD with mortality and sputum microbiological status.

Outcomes	CAD^+^ AMI^-^				CAD^+^ AMI^+^				ATS			
	Unadjusted Effect size (95%CI)	*P*-value	Adjusted Effect size (95%CI) ^#&^	*P*-value	Unadjusted Effect size (95%CI)	*P*-value	Adjusted Effect size (95%CI) ^#&^	*P*-value	Unadjusted Effect size (95%CI)	*P*-value	Adjusted Effect size (95%CI) ^#$^	*P*-value
All-cause mortality (HR)	1.73 (1.29–2.32)	< 0.001	1.31 (0.91–1.89)	0.151	2.24 (1.62–3.11)	< 0.001	2.04 (1.38–3.00)	< 0.001	2.99 (2.21–4.06)	< 0.001	2.79 (1.92–4.07)	< 0.001
Infection-related (HR) mortality	1.73 (1.17–2.56)	0.006	1.36 (0.84–2.21)	0.205	2.34 (1.53–3.58)	< 0.001	1.95 (1.17–3.24)	0.011	2.79 (1.84–4.23)	< 0.001	2.04 (1.19–3.46)	0.009
Sputum Culture positivity (OR)	0.62 (0.33–1.18)	0.148	0.75 (0.37–1.54)	0.438	1.57 (0.85–2.90)	0.150	1.24 (0.59–2.58)	0.562	0.89 (0.42–1.93)	0.785	0.71 (0.26–1.92)	0.495
Sputum AFB smear positivity (OR)	0.51 (0.19–1.42)	0.199	0.95 (0.31–2.94)	0.926	1.14 (0.45–2.91)	0.781	1.29 (0.46–3.67)	0.627	1.03 (0.37–2.91)	0.951	1.84 (0.49–6.75)	0.360

AFB: Acid-fast bacilli; ATS: Atherothrombotic stroke, CAD + AMI + : Coronary artery disease with history of Acute myocardial infarction; CAD + AMI-: Coronary artery disease without history of Acute myocardial infarction, HR: Hazard ratio, OR: Odds ratio.

^#^Adjusted for age, gender, body mass index (BMI), diabetes mellitus (DM), hypertension (HTN), cancer, chronic kidney disease (stage 3–5), asthma, chronic obstructive pulmonary disease (COPD), liver cirrhosis, transplant status, baseline sputum AFB status, cavitary disease at baseline, metformin use, statin use, and calcium channel blocker use.

^&^ Additionally adjusted for ATS.

^$^ Additionally adjusted for CAD.

#### Microbiological outcomes

After adjusting for confounders, the CAD^+^AMI^−^ group had an odds ratio (OR) of 0.75 (95%CI, 0.37–1.54) and the CAD^+^AMI^+^ group had a OR of 1.24 (95%CI, 0.59–2.58) for sputum culture positivity at 2 months, compared to the non-CAD group. After adjusting for confounders, the CAD^+^AMI^−^ group had an OR of 0.95 (95%CI, 0.31–2.94) and the CAD^+^AMI^+^ group had an OR of 1.29 (95%CI, 0.46–3.67) for sputum smear positivity at 2 months compared to the non-CAD group (Table 2).

### Stratification based on ATS

#### Patient characteristics

Compared to the non-ATS group, patients with ATS were older (median age: 65.9 vs. 77.8 years, *P* < 0.001), were equally likely to be male (68.4% vs. 63.7%, *P* = 0.318), and had a similar BMI (mean BMI (kg/m^2^): 21.3 vs. 21.5, *P* = 0.545). Sputum AFB smear status at baseline was similar in the two groups (Table 1). The non-ATS group had a higher proportions of patients with cavitary TB disease at baseline (14.5% vs 7% *P* = 0.034) compared to patients with ATS (Table 1).

#### Mortality

There was a significantly higher all-cause mortality in the ATS group (45/95 patients, 44.1%) compared to the non-CAD group (499/2572 patients, 17.9%) (*P* < 0.001) by χ^2^ test (Table 1). The log-rank test Kaplan–Meier analysis showed that patients in the ATS group had shorter time to all-cause and infection-related mortality compared to patients without ATS (Fig. 2C,D; *P* < 0.001). After adjusting for confounders, patients in the ATS group had a HR of 2.79 (95%CI 1.92–4.07) for all-cause mortality and 2.04 (95%CI, 1.19–3.46) for infection-related mortality (Table 2).

#### Microbiological outcomes

After adjusting for confounders, the ATS group had an OR of 0.71 (95%CI, 0.26–1.92) for sputum culture positivity at 2 months and an OR of 1.84 (95%CI, 0.49–6.75) for sputum smear positivity at 2 months compared to the non-ATS group (Table 2).

### Association of ASCVD with systemic inflammation and its mediation of mortality

After adjusting for confounders, the CAD^+^AMI^+^ group had a significantly higher mean CRP (Regression co-efficient B (SE), 1.24 (0.62), *P* = 0.040) and the CAD^+^AMI^−^ group had a non-significantly higher CRP (B (SE), 1.04(0.60), *P* = 0.087) compared to the non-CAD group in the linear regression analysis (Table 3). The ATS group had a non-significantly higher CRP (B (SE), 0.49(0.68), *P* = 0.466) compared to the non-ATS group at baseline. No similar association was noted between the presence of CAD or ATS and other inflammatory markers, such as WBC or NL ratio at baseline.

**Figure 3:**
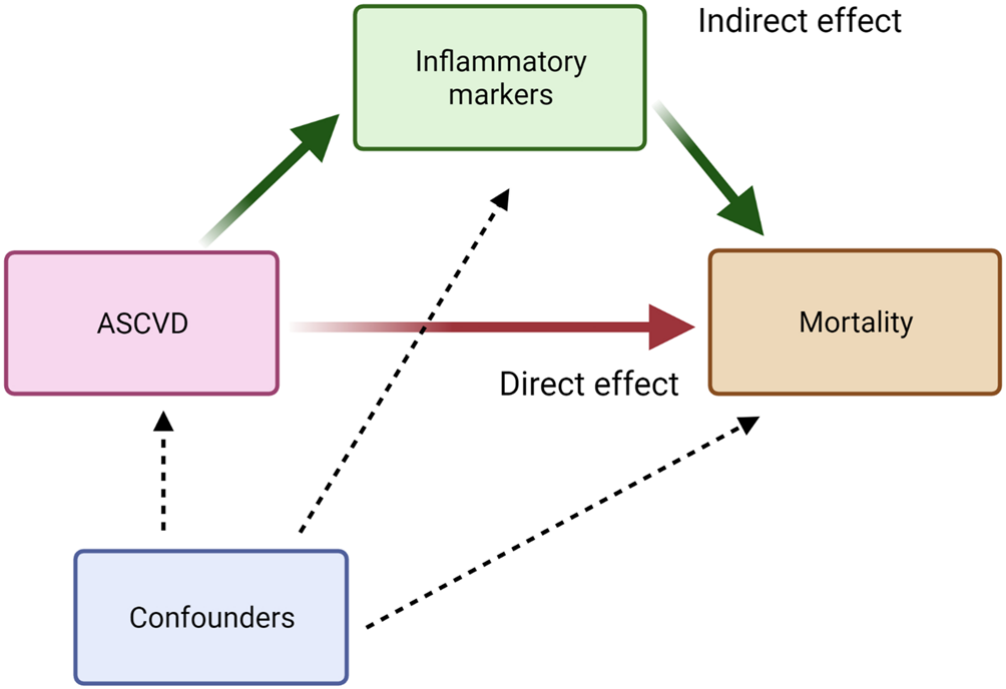
Causal directed acyclic graph (cDAG) for the analysis of the mediation of the association of Atherosclerotic cardiovascular disease (ASCVD) with mortality by inflammatory markers. Exposure: ASCVD: CAD^+^AMI^−^, CAD^+^AMI^+^ and ATS were assessed in separate models. Mediator: Inflammatory markers: CRP, WBC and NL ratio were assessed in separate models Outcomes: All-cause and infection related mortality were assessed in separate models. Confounders: Age, gender, body mass index, diabetes mellitus, hypertension, cancer, chronic kidney disease (stage 3–5), asthma, chronic obstructive pulmonary disease, liver cirrhosis, transplant status, baseline sputum acid fast bacilli status, cavitary disease at baseline, metformin use, statin use, and calcium channel blocker use. ATS, pre-existing atherothrombotic stroke; CAD, pre-existing coronary artery disease; CAD^+^AMI^−^, Pre-existing coronary artery disease without prior acute myocardial infarction; CAD^+^AMI^+^, Pre-existing coronary artery disease with prior acute myocardial infarction. CRP: C-reactive protein; NL ratio: Neutrophil lymphocyte ratio; WBC: Total leukocyte count (× 10^3^/µL).

The causal directed acyclic graph (cDAG) representing our proposed mediation hypothesis linking the exposure (pre-existing ASCVD) and the outcome (all-cause and infection-related mortality during TB treatment) by using inflammatory markers (measured by CRP, NL ratio, WBC count) as the potential mediators is shown in Fig. 3. CRP mediated 66% of the association of CAD^+^AMI^−^ with all-cause mortality (*P* = 0.048) and 73% of the association with infection-related mortality (*P* = 0.035) (Table 4). CRP mediated 25% of the association of CAD^+^AMI^+^ with all-cause mortality (*P* = 0.033) and 36% of the association with infection-related mortality (*P* = 0.042). CRP did not significantly mediate the association of ATS with all-cause or infection-related mortality (Table 4). WBC and NL ratio were not found to mediate the association of ASCVD and mortality.

**Table 3:** Association of CAD and acute myocardial infarction (AMI) with inflammatory parameters.

Inflammatory markers	CAD^+^ AMI^-^				CAD^+^ AMI^+^				ATS			
	Unadjusted B(SE)	*P*-value	Adjusted B(SE) ^#&^	*P*-value	Unadjusted B(SE)	*P*-value	Adjusted B(SE) ^#&^	*P*-value	B(SE)	*P*-value	AdjustedB(SE) ^#$^	*P*-value
C-reactive protein	1.08 (0.52)	**0.038**	1.04 (0.60)	**0.087**	1.41 (0.57)	**0.038**	1.24 (0.62)	**0.040**	**0.61 (0.61)**	**0.315**	**0.49 (0.68)**	**0.466**
WBC	0.19 (0.33)	**0.567**	0.22 (0.38)	**0.563**	-0.17 (0.41)	**0.670**	-0.00(0.44)	**0.993**	**0.12 (0.43)**	**0.783**	**0.12 (0.49)**	**0.801**
NL ratio	0.03 (0.19)	**0.891**	-0.16 (0.21)	**0.452**	0.20 (0.23)	**0.387**	0.22 (0.23)	**0.346**	**0.56 (0.24)**	**0.018**	**0.40 (0.26)**	**0.127**

ATS: Atherothrombotic stroke, CAD + AMI + : Coronary artery disease with history of Acute myocardial infarction; CAD + AMI-: Coronary artery disease without history of Acute myocardial infarction, B: Regression co-efficient, SE: Standard error.

^#^Adjusted for age, gender, body mass index (BMI), diabetes mellitus (DM), hypertension (HTN), cancer, chronic kidney disease (stage 3–5), asthma, chronic obstructive pulmonary disease (COPD), liver cirrhosis, transplant status, baseline sputum AFB status, cavitary disease at baseline, metformin use, statin use, and calcium channel blocker use.

^&^Additionally adjusted for ATS.

^$^Additionally adjusted for CAD.

**Table 4:** Assessment of mediation of the effect of CAD and ATS on mortality by inflammatory parameters (adjusted analysis).

Outcome	All-cause mortality				Infection-related mortality			
	Total effect β (95%CI)	Direct effect β (95%CI)	Indirect effect β (95%CI)	Effect mediated by inflammatory parameters % (95%CI)	Total effect β (95%CI)	Direct effect β (95%CI)	Indirect effect β (95%CI)	Effect mediated by inflammatory parameters% (95%CI)
**Effect Mediation by CRP**								
**CAD**^**+**^** AMI**^**-**^	0.05 (-0.07 to 0.17) (*P* = 0.439)	0.02 (-0.09 to 0.13) (*P* = 0.407)	0.03(0.01 to 0.06) (*P* = 0.047)	66% (*P* = 0.048)	0.03 (-0.07 to 0.14) (*P* = 0.559)	0.01 (-0.09 to 0.11) (*P* = 0.876)	0.02 (0.01 to 0.04) (*P* = 0.035)	73% (*P* = 0.035)
**CAD**^**+**^** AMI**^**+**^	0.13 (0.01 to 0.23) (*P* = 0.038)	0.09 (-0.02 to 0.21) (*P* = 0.111)	0.03 (0.01 to 0.06) (*P* = 0.033)	25% (*P* = 0.033)	0.07 (-0.03 to 0.18) (*P* = 0.180)	0.05 (-0.04 to 0.13) (*P* = 0.297)	0.03 (0.01 to 0.05) (*P* = 0.033)	36% (*P* = 0.042)
ATS	0.07 (-0.05 to 0.19) (*P* = 0.257)	0.07 (-0.04 to 0.18) (*P* = 0.259)	0.01 (-0.02 to 0.04) (*P* = 0.814)	6% (0.828)	0.04(-0.07 to 0.15) (*P* = 0.459)	0.04 (-0.07 to 0.15) (*P* = 0.494)	0.01 (-0.02 to 0.03) (*P* = 0.812)	7% (*P* = 0.829)
**Effect Mediation by WBC**								
**CAD**^**+**^** AMI**^**-**^	0.03 (-0.05 to 0.11) (*P* = 0.422)	0.04 (-0.05 to 0.10) (*P* = 0.531)	0.01 (-0.01 to 0.02) (*P* = 0.223)	25% (*P* = 0.226)	0.03 (-0.04 to 0.09) (*P* = 0.362)	0.03 (-0.04 to 0.09) (*P* = 0.427)	0.01(-0.01 to 0.02) (*P* = 0.262)	15% (0.258)
**CAD**^**+**^** AMI**^**+**^	0.13 (0.04 to 0.22) (*P* = 0.010)	0.13 (0.03 to 0.22) (*P* = 0.010)	-0.01 (-0.02 to 0.02) (*P* = 0.796)	1% (*P* = 0.796)	0.08 (-0.01 to 0.16) (*P* = 0.065)	0.08 (-0.01 to 0.16) (*P* = 0.058)	-0.01 (-0.01 to 0.01) (*P* = 0.796)	1% (0.789)
ATS	0.20 (0.09 to 0.31) (*P* < 0.001)	0.20 (0.09 to 0.30) (*P* < 0.001)	0.01 (-0.02 to 0.02) (*P* = 0.714)	1% (*P* = 0.728)	0.07 (-0.02 to 0.16) (*P* = 0.124)	0.07 (-0.02 to 0.16) (*P* = 0.128)	0.01 (-0.01 to 0.02) (*P* = 0.716)	2% (*P* = 0.736)
**Effect Mediation by NL ratio**								
**CAD**^**+**^** AMI**^**-**^	0.04 (-0.05 to 0.12) (*P* = 0.416)	0.05 (-0.04 to 0.12) (*P* = 0.333)	-0.01 (-0.03 to 0.01) (*P* = 0.590)	14% (*P* = 0.581)	0.03 (-0.03 to 0.11) (*P* = 0.364)	0.04 (-0.04 to 0.11) (*P* = 0.317)	-0.01 (-0.02 to 0.01) (*P* = 0.594)	8% (*P* = 0.586)
**CAD**^**+**^** AMI**^**+**^	0.12 (0.02 to 0.21) (*P* = 0.006)	0.11 (0.01 to 0.21) (*P* = 0.031)	0.01 (-0.02 to 0.03) (*P* = 0.492)	7% (*P* = 0.512)	0.07 (-0.01 to 0.15) (*P* = 0.104)	0.07 (-0.02 to 0.15) (*P* = 0.131)	0.01 (-0.01 to 0.02) (*P* = 0.491)	8% (*P* = 0.517)
ATS	0.19 (0.08 to 0.30) (*P* = 0.001)	0.18 (0.07 to 0.29) (*P* = 0.002)	0.01 (-0.02 to 0.04) (*P* = 0.440)	6% (*P* = 0.452)	0.06 (-0.03 to 0.16) (*P* = 0.180)	0.06 (-0.04 to 0.15) (*P* = 0.234)	0.01 (-0.01 to 0.02) (*P* = 0.429)	10% (*P* = 0.459)

ATS: Atherothrombotic stroke, CAD + AMI + : Coronary artery disease with history of Acute myocardial infarction; CAD + AMI-: Coronary artery disease without history of Acute myocardial infarction, B: Regression co-efficient, SE: Standard error.

^#^Adjusted for age, gender, body mass index (BMI), diabetes mellitus (DM), hypertension (HTN), cancer, chronic kidney disease (stage 3–5), asthma, chronic obstructive pulmonary disease (COPD), liver cirrhosis, transplant status, metformin use, statin use, and calcium channel blocker use.

^&^Additionally adjusted for ATS.

^$^Additionally adjusted for CAD.

### Stratification based on statin use among patients with ASCVD (CAD or ATS)

#### Patient characteristics

Among 355 patients with pre-existing ASCVD, 65 patients (18.3%) received statins according to the intention to treat definition and 45 patients (12.7%) received statins according to the per-protocol definition. Patient characteristics stratified by statin use according to the intention-to-treat exposure are shown in Table 5.

**Table 5:** Patient characteristics stratified by statin user in patients with cardiovascular disorders.

Study characteristics	Measure	Total (N = 355)	Statin non-users (n = 290)	Statin users (n = 65)	*P*-value
Age (years)	Median (IQR)	76.7 (69.1–84.1)	77.1 (69.1–84.4))	75.8 (67.7–83.5)	0.395
Male Sex	No (%)	260 (73.2%)	207 (71.4%)	53 (81.5%)	0.094
BMI	Mean (SD)	21.8 (0.22)	21.5 (0.24)	23.2 (0.48)	0.002
Smoking	No. (%)	130(41.3%)	99(39.3%)	31(49.2%)	0.153
Alcoholism	No. (%)	9 (2.5%)	8 (2.8%)	1 (1.5%)	0.572
DM	No. (%)	124(34.9%)	91(31.4%)	33(50.8%)	0.003
HTN	No. (%)	263(74.1%)	207(71.4%)	56(86.2%)	0.014
Cancer	No. (%)	73(20.6%)	63(21.7%)	10(15.4%)	0.253
CKD Stage ≥ 3	No. (%)	141(39.7%)	112(38.6%)	29(44.6%)	0.372
Asthma	No. (%)	20(5.6%)	19(6.6%)	1(1.5%)	0.113
COPD	No. (%)	98(27.6%)	79(27.2%)	19(29.2%)	0.746
Bronchiectasis	No. (%)	17(4.8%)	13(4.5%)	4(6.2%)	0.568
Pneumoconiosis	No. (%)	5(1.4%)	3(1.0%)	2(3.1%)	0.207
Liver cirrhosis	No. (%)	6(1.7%)	6(2.1%)	0(0%)	0.242
History of transplant	No. (%)	7(1.9%)	6(2.1%)	1(1.5%)	0.781
HIV	No. (%)	4(1.1%)	3(1.0%)	1(1.5%)	0.728
CAD	No. (%)	291(81.9%)	232(80%)	59(90.8%)	0.041
ATS	No. (%)	102(28.7%)	86(29.7%)	16(24.6%)	0.417
Baseline lipid levels					
LDL	Mean (SD)	86.1 (5.2)	83.5 (24.8)	89.6 (38.1)	0.572
HDL	Mean (SD)	37.3 (2.5)	39.5 (19.6)	34.3 (2.8)	0.314
TCHOL	Mean (SD)	150.3 (4.7)	149.5 (6.2)	151.5 (7.3)	0.836
TG	Mean (SD)	96.8 (6.8)	86.3 (8.6)	119.3 (9.8)	0.024
TB disease characteristics					
Initial AFB smear positivity	No. (%)	144(42.1%)	113(40.2%)	31(50.8%)	0.128
Initial AFB smear grade	Median (IQR)	0 (0–2)	0 (0–2)	1 (0–2)	0.111
Prior TB	No. (%)	16(7.4%)	11(6.1%)	5(13.5%)	0.117
Cavitary disease	No. (%)	28(7.9%)	22(7.6%)	6(9.2%)	0.657
Cardiovascular drug use					
Metformin users	No. (%)	55(15.5%)	35(12.1%)	20(30.8%)	< 0.001
Calcium channel blocker users	No. (%)	107(30.1%)	84(28.9%)	23(35.4%)	0.308
Outcome					
All-cause mortality	No. (%)	115 (34.5%)	102 (37.9%)	13 (20.3%)	0.008
Infection-related mortality	No. (%)	67 (20.1%)	59 (21.9%)	8 (12.5%)	0.091

AFB: Acid-fast bacilli; BMI: Body mass index; ATS: Atherothrombotic stroke, CAD: Coronary artery disease; CAD + AMI + : Coronary artery disease with history of Acute myocardial infarction; CAD + AMI-: Coronary artery disease without history of Acute myocardial infarction; CCB: Calcium channel blocker; CCI: Charlson comorbidity index; CHF: Congestive heart failure; CKD: Chronic kidney disease; COPD: Chronic obstructive pulmonary disease; CVA: Cerebrovascular accident; DM: Diabetes mellitus; HDL: High density lipoprotein cholesterol; HIV: Human immunodeficiency virus; HTN: Hypertension; IQR, Interquartile range; LDL: Low density lipoprotein cholesterol; PVD: Peripheral vascular disease; SD: Standard deviation.

#### Mortality

**Figure 4:**
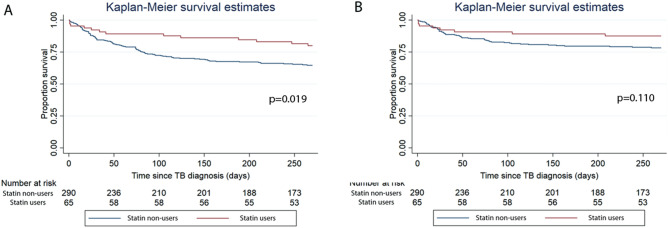
Kaplan Meier analysis on the association of statin use among patients with Atherosclerotic cardiovascular disease (ASCVD) with (**A**) all-cause mortality and (**B**) infection-related mortality.

Patients receiving statins (as per the intention-to treat definition) had a HR of 0.41 (95%CI, 0.19–0.84) for all-cause mortality and a HR of 0.42 (95%CI, 0.17–1.06) for infection-related mortality after adjusting for confounders (Table 6, Fig. 4A,B). Using the per-protocol definition, patients receiving statins had a HR of 0.41 (95%CI, 0.18–0.96) for all-cause mortality and a HR of 0.43 (95%CI, 0.15–1.25) for infection-related mortality after adjusting for confounders (Table 6). Sensitivity analyses excluding patients who died less than 30 days from treatment onset (Supplementary Table 1) and excluding patients who did not receive the required dose of statin from the comparison group (Supplementary Table 2) showed lower hazard for all-cause mortality but not infection-related mortality among statin users in the intention-to-treat analysis.

**Table 6:** Association of statin use with mortality in patients with cardiovascular disorders.

Characteristic	Statin Use (Intention-to-treat)						Statin Use (Per-protocol)					
	Unadjusted HR	(95%CI)	*P*-value	Adjusted HR#	95%CI	*P*-value	Unadjusted HR	(95%CI)	*P*-value	Adjusted HR#	95%CI	*P*-value
All-cause mortality	0.51	0.28–0.90	**0.021**	0.41	0.19–0.84	**0.015**	**0.46**	**0.23–0.95**	**0.036**	**0.41**	**0.18–0.96**	**0.041**
Infection-related mortality	0.55	0.26–1.16	**0.116**	0.42	0.17–1.06	**0.065**	0.55	0.26–1.16	**0.116**	**0.43**	**0.15–1.25**	**0.122**

^#^Adjusted for age, gender, body mass index (BMI), diabetes mellitus (DM), hypertension (HTN), cancer, chronic kidney disease (stage 3–5), asthma, chronic obstructive pulmonary disease (COPD), liver cirrhosis, transplant status, baseline sputum AFB status, cavitary disease at baseline, metformin use, and calcium channel blocker use.

## Discussion

Our retrospective cohort study found that a history of prior AMI and ATS was associated with higher all-cause and infection-related mortality during the first nine months after TB treatment initiation independent of confounding factors despite the comparison group having higher rates of cavitary TB disease at baseline. Pre-existing CAD (AMI^−^ or AMI^+^) or ATS was not associated with microbiological outcomes, namely sputum culture conversion or sputum smear AFB positivity at two months of TB treatment. The CAD^+^AMI^−^ and CAD^+^AMI^+^ groups had significantly higher levels of systemic inflammation at TB diagnosis, manifested by higher CRP levels in the univariable analysis. The multivariable analysis showed significant association only for the CAD^+^AMI^+^ group, suggesting a relationship between the progression of CAD with systemic levels of inflammation. Higher NL ratios were noted in the ATS group only in univariable, but not multivariable, analysis.

TB and cardiovascular diseases have a close epidemiological and geographical overlap^36^. An important relationship has been demonstrated in the pathogenesis of TB and ASCVD^37^. The key hallmarks of atherosclerosis are LDL oxidation, foam cell formation and inflammation^38^. Preclinical studies have shown that Mtb infection of guinea pigs resulted in systemic oxidative stress, depletion of serum total antioxidant capacity and accumulation of oxidized LDL in granulomas^39^, with an increased expression of key atherosclerosis markers, such as scavenger receptor CD36 and lectin oxidized LDL receptor 1^39^.

A systematic review indicated that patients with TB have an increased risk of CAD^4^. Also, pulmonary TB was associated with a higher hazard of progression to AMI in a propensity score-matched analysis from a US insurance claims database^7^. Likewise, patients with TB have an increased risk of ischemic^9,10^, but not hemorrhagic stroke in the first three years after TB diagnosis^10^ when compared to non-infected controls. Multiples cases of TB causing coronary arteritis and subsequent AMI have been reported^40,41^. Preclinical studies have shown that mycobacterial infection aggravates atherosclerosis formation in the aortas of hyperlipidemic, lipoprotein receptor knockout (*Ldlr*^−/−^) mice^42^. These data suggest that *M. tuberculosis* and associated systemic inflammation may play a causative role in atherosclerosis.

Prior reports in non-TB populations have shown that higher levels of systemic inflammation increase plaque instability, resulting in plaque rupture, fissuring, or erosion^14,43–46^. In particular, patients with CAD and elevated CRP levels have higher rates of death and progression to AMI, relative to patients with lower CRP^47,48^. Our study demonstrated the consistency of these findings in ASCVD patients with TB as well, with CAD patients (AMI^−^ or AMI^+^) having greater levels of baseline CRP compared to patients without CAD. Similarly, patients with a history of ATS were found to have higher NL ratios than patients without ATS. Higher levels of inflammatory markers, such as CRP, WBC, and NL ratio, have been associated with a poor prognosis in patients with TB^8,18^. Our mediation analysis assessed whether the higher mortality observed in the CAD and ATS groups was mediated by systemic inflammation. We found that more than nearly one-quarter of the hazard of mortality in TB patients with AMI is mediated by systemic inflammation, as reflected by elevated serum CRP levels. These findings raise the intriguing possibility of targeting systemic inflammation as a potential intervention to reduce mortality and improve outcomes among TB patients with ASCVD.

Statins are commonly used agents used to reduce serum lipids and systemic inflammation to improve outcomes in patients with a history of or at risk for ASCVD. Prior systematic reviews have shown that statin use reduces TB incidence, both in patients with DM and in the general population^28,29^. Preclinical studies in macrophage and mouse models have demonstrated that statins enhance autophagy and phagosome maturation^24^, increase the proportion of NK cells, and increase secretion of the pro-inflammatory cytokines IL-1β and IL-12p70, thereby reducing the lung bacillary burden in *M. tuberculosis*-infected mice^24,49^. We found that the anti-tubercular activity of statins represents a class effect^50^, which is mediated by inhibition of cholesterol biosynthesis and autophagy via the AMPK-mTORC1-TFEB axis in macrophages^25^. These studies demonstrate that statins enhance the anti-tubercular activity of the first-line regimen and activate the host immune response against *M. tuberculosis*. Although a cohort study from an insurance claims database showed that TB treatment completion rates did not improve following statin therapy^30^, the study did not appropriately adjust for confounding factors. Furthermore, no previous clinical study has evaluated the effect of statin use on important TB-related outcomes, such as long-term lung function or sputum microbiological outcomes^51^. In our retrospective cohort, among patients with ASCVD, statin users had a lower hazard of all-cause mortality. We did not note a similar significant association between statin use and infection-related mortality. Though our cohort included only drug-susceptible TB patients, we believe that statin therapy may have similar beneficial effects in drug-resistant TB as well.

With respect to the strengths of our study, our large sample size enabled adjusting for multiple confounders. Availability of data on the serum inflammatory markers in a relatively large number of patients at baseline enabled mediation analysis to assess the effect of CAD mediated by systemic inflammation. Our study also has a few limitations. Follow-up levels of serum inflammatory markers following initiation of TB treatment would have enabled a trend of these parameters in patients with or without systemic inflammation at baseline. We did not have data on aspirin use, which can act as a confounding factor. We did not have a control population with respiratory or non-respiratory infections other than TB in our cohort to further assess the role played by systemic inflammation in cardiovascular diseases. The relatively small sample size of patients receiving statin therapy prevented an analysis of the effect of statin use on sputum smear and culture results.

In summary, patients with CAD and ATS have higher hazards of all-cause and infection-related mortality during the first nine months after TB treatment initiation. Elevated serum inflammation markers (CRP) mediate nearly one-quarter to one-third of this association when adjusted for confounders. However, pre-existing CAD or ATS was not associated with a difference in sputum culture or smear positivity at two months. Statin use was associated with lower all-cause mortality among patients with ASCVD. Randomized controlled trials are required to assess the utility of adjunctive statin therapy on microbiological and clinical outcomes in TB patients with and without ASCVD.

## Supplementary Information

Supplementary Information 1.

Supplementary Information 2.

## References

[1] Tuberculosis. Available at: https://www.who.int/news-room/fact-sheets/detail/tuberculosis. Accessed 22 February 2021.

[2] Yusuf S (2014). Cardiovascular risk and events in 17 low-, middle-, and high-income countries. N. Engl. J. Med..

[3] Bowry ADK, Lewey J, Dugani SB, Choudhry NK (2015). The burden of cardiovascular disease in low- and middle-income countries: epidemiology and management. Can. J. Cardiol..

[4] Wongtrakul W, Charoenngam N, Ungprasert P (2020). Tuberculosis and risk of coronary heart disease: a systematic review and meta-analysis. Indian J. Tuberc..

[5] Alsayed HAF (2018). Latent tuberculosis infection among patients with coronary artery stenosis: a case-control study. Int. J. Mycobacteriol..

[6] Huaman MA (2021). Latent tuberculosis infection and subclinical coronary atherosclerosis in Peru and Uganda. Clin. Infect. Dis..

[7] Huaman MA (2017). Tuberculosis and risk of acute myocardial infarction: a propensity score-matched analysis. Epidemiol. Infect..

[8] Huaman MA (2018). The relationship between latent tuberculosis infection and acute myocardial infarction. Clin. Infect. Dis..

[9] Salindri AD, Wang JY, Lin HH, Magee MJ (2019). Post-tuberculosis incidence of diabetes, myocardial infarction, and stroke: retrospective cohort analysis of patients formerly treated for tuberculosis in Taiwan, 2002–2013. Int. J. Infect. Dis..

[10] Sheu JJ, Chiou HY, Kang JH, Chen YH, Lin HC (2010). Tuberculosis and the risk of ischemic stroke: a 3-year follow-up study. Stroke.

[11] Basham CA, Smith SJ, Romanowski K, Johnston JC (2020). Cardiovascular morbidity and mortality among persons diagnosed with tuberculosis: a systematic review and meta-analysis. PLoS ONE.

[12] Pai JK (2004). Inflammatory markers and the risk of coronary heart disease in men and women. N. Engl. J. Med..

[13] Rader DJ (2000). Inflammatory markers of coronary risk. N. Engl. J. Med..

[14] Liu Y (2020). Impact of high-sensitivity C-reactive protein on coronary artery disease severity and outcomes in patients undergoing percutaneous coronary intervention. J. Cardiol..

[15] Chen H (2019). Monocyte/lymphocyte ratio is related to the severity of coronary artery disease and clinical outcome in patients with non-ST-elevation myocardial infarction. Med. (United States).

[16] Whiteley W (2009). Inflammatory markers and poor outcome after stroke: a prospective cohort study and systematic review of interleukin-6. PLoS Med..

[17] Mesquita EDD (2016). Associations between systemic inflammation, mycobacterial loads in sputum and radiological improvement after treatment initiation in pulmonary TB patients from Brazil: a prospective cohort study. BMC Infect. Dis..

[18] Singanayagam A (2016). Evaluation of serum inflammatory biomarkers as predictors of treatment outcome in pulmonary tuberculosis. Int. J. Tuberc. Lung Dis..

[19] Wilson D (2018). Evaluation of tuberculosis treatment response with serial C-reactive protein measurements. Open Forum Infect. Dis..

[20] Cudahy PGT, Warren JL, Cohen T, Wilson D (2018). Trends in C-reactive protein, D-dimer, and fibrinogen during therapy for HIV-associated multidrug-resistant tuberculosis. Am. J. Trop. Med. Hyg.

[21] Lawn SD, Kerkhoff AD, Vogt M, Wood R (2013). Diagnostic and prognostic value of serum C-reactive protein for screening for HIV-associated tuberculosis. Int. J. Tuberc. Lung Dis..

[22] Kim CW (2012). Risk factors related with mortality in patient with pulmonary tuberculosis. Tuberc. Respir. Dis. (Seoul).

[23] Jain MK, Ridker PM (2005). Anti-inflammatory effects of statins: clinical evidence and basic mechanisms. Nat. Rev. Drug Discov..

[24] Parihar SP (2014). Statin therapy reduces the mycobacterium tuberculosis burden in human macrophages and in mice by enhancing autophagy and phagosome maturation. J. Infect. Dis..

[25] Bruiners N (2020). The anti-tubercular activity of simvastatin is mediated by cholesterol-driven autophagy via the AMPK-mTORC1-TFEB axis. J. Lipid Res..

[26] Dutta NK (2016). Statin adjunctive therapy shortens the duration of TB treatment in mice. J. Antimicrob. Chemother..

[27] Dutta NK (2020). Adjunctive host-directed therapy with statins improves tuberculosis-related outcomes in mice. J. Infect. Dis..

[28] Duan H, Liu T, Zhang X, Yu A, Cao Y (2020). Statin use and risk of tuberculosis: a systemic review of observational studies. Int. J. Infect. Dis..

[29] Li X, Sheng L, Lou L (2020). Statin use may be associated with reduced active tuberculosis infection: a meta-analysis of observational studies. Front. Med..

[30] Chen YT, Kuo SC, Chao PW, Chang YY (2019). Use of lipid-lowering agents is not associated with improved outcomes for tuberculosis patients on standard-course therapy: a population-based cohort study. PLoS ONE.

[31] Nahid P (2016). Official American thoracic society/centers for disease control and prevention/infectious diseases society of America clinical practice guidelines: treatment of drug-susceptible tuberculosis. Clin. Infect. Dis..

[32] Chidambaram V, Gupte A, Wang J-Y, Golub JE, Karakousis PC (2020). The impact of hypertension and use of calcium channel blockers on tuberculosis treatment outcomes. Clin. Infect. Dis..

[33] Grundy SM (2019). 2018 AHA/ACC/AACVPR/AAPA/ABC/ACPM/ADA/AGS/APhA/ASPC/NLA/PCNA guideline on the management of blood cholesterol: a report of the American college of cardiology/American heart association task force on clinical practice guidelines. Circulation.

[34] Mehmetoglu M (2018). Medsem: a Stata package for statistical mediation analysis. Int. J. Comput. Econ. Econom..

[35] Zhao X, Lynch JG, Chen Q (2010). Reconsidering Baron and Kenny: myths and truths about mediation analysis. J. Consum. Res..

[36] Huaman MA, Henson D, Ticona E, Sterling TR, Garvy BA (2015). Tuberculosis and cardiovascular disease: linking the epidemics. Trop. Dis. Travel Med. Vaccin..

[37] Venketaraman V (2010). Atherosclerosis: pathogenesis and increased occurrence in individuals with HIV and Mycobacterium tuberculosis infection. HIV/AIDS Res. Palliat. Care.

[38] Antonov AS, Kolodgie FD, Munn DH, Gerrity RG (2004). Regulation of macrophage foam cell formation by αVβ3 integrin: potential role in human atherosclerosis. Am. J. Pathol..

[39] Palanisamy GS (2012). Uptake and accumulation of oxidized low-density lipoprotein during mycobacterium tuberculosis infection in guinea pigs. PLoS ONE.

[40] Kinare SG, Bhatia BI (1971). Tuberculous coronary arteritis with aneurysm of the ventricular septum. Chest.

[41] Chan S (2018). An unusual case of mycobacterium tuberculous coronary arteritis and thrombosis resulting in acute myocardial infarction. Forensic. Sci. Med. Pathol..

[42] Huaman MA (2020). Mycobacterium bovis Bacille-Calmette-Guérin infection aggravates atherosclerosis. Front. Immunol..

[43] Zakynthinos E, Pappa N (2009). Inflammatory biomarkers in coronary artery disease. J. Cardiol..

[44] Bhat T (2013). Neutrophil to lymphocyte ratio and cardiovascular diseases: a review. Expert Rev. Cardiovasc. Ther..

[45] Shah PK (2002). Pathophysiology of coronary thrombosis: Role of plaque rupture and plaque erosion. Prog. Cardiovasc. Dis..

[46] Hansson GK, Libby P, Tabas I (2015). Inflammation and plaque vulnerability. J. Intern. Med..

[47] Niccoli G (2008). Independent prognostic value of C-reactive protein and coronary artery disease extent in patients affected by unstable angina. Atherosclerosis.

[48] Toss H, Lindahl B, Siegbahn A, Wallentin L (1997). Prognostic influence of increased fibrinogen and C-reactive protein levels in unstable coronary artery disease. Circulation.

[49] Guerra-De-Blas PDC (2019). Simvastatin enhances the immune response against mycobacterium tuberculosis. Front. Microbiol..

[50] Dutta, N. & Karakousis, P. Statins as host-directed therapy for tuberculosis. In *Advances in Host-Directed Therapies Against Tuberculosis* (eds. Karakousis, P. C., Hafner, R. & Laura, M.) (Springer, 2021). 10.1007/978-3-030-56905-1.

[51] Chidambaram V, Karakousis PC (2021). Reply to Lai et al.. J. Infect. Dis..

