# Supplementary material for: The association of atherosclerotic cardiovascular disease and statin use with inflammation and treatment outcomes in tuberculosis
Source: Sci Rep. 2021 Jul 27;11:15283. doi: 10.1038/s41598-021-94590-x (PMC8316554; doi:10.1038/s41598-021-94590-x)
Supplement: Supplementary file 2 — Supplementary Information 2. [file 41598_2021_94590_MOESM2_ESM.pdf]

SUPPLEMENTARY DOCUMENT

Supplementary Methods

Patient characteristics, stratified into three groups of CAD status (non-CAD, CAD^+AMI^-, CAD^+AMI^+), were compared using ANOVA for normally distributed data, Kruskal-Wallis test for non-normally distributed data, and chi-square (χ^2) test for categorical variables. Patient characteristics stratified by the presence of ATS, the second exposure of interest, were compared using two-sided t-test and χ^2 test for continuous and categorical variables, respectively. Kaplan-Meier analysis and Cox proportional hazards regression was used to measure the association between the above exposures and all-cause and infection-related mortality in separate models. Detailed methods are described in the supplementary document (section I). Person-time at risk of outcome stratified by the above exposures, was calculated from the time of TB treatment initiation until 9 months, or loss to follow-up or death, whichever occurred first. Point of loss to follow-up was defined as the last study visit prior to 9 months. The association between the above exposures and sputum-smear and culture positivity at 2 months were analyzed using univariable and multivariable logistic regression. Potential confounders for multivariable analyses were identified by literature review and by exploratory univariable data analysis at $p < 0.05$ significance, depending on the exposure that was assessed.

The association of pre-existing CAD and ATS, and serum inflammatory markers, namely CRP, WBC and NL ratio, was analyzed using univariable and multivariable linear regression analyses. We performed sensitivity analysis to assess this association by including the results of inflammatory markers tested only up to day 15 of TB treatment to account for the possibility of a rapid decrease in inflammation after initiation of TB treatment. We assessed the potential mediation by the levels of inflammation for the association between ASCVD and all-cause and infection-related mortality in patients with TB. We constructed a causal directed acyclic graph(cDAG) to represent our proposed mediation hypothesis linking the exposure (pre-existing CAD or ATS) using inflammatory markers (measured by CRP, NLratio, WBC count) as the potential mediators and all-cause and infection-related mortality during TB treatment as the outcome with the potential confounders identified above. We estimated the path coefficients using the structural equation modelling (SEM). The effect on ASCVD on mortality that is mediated through the inflammatory markers was

considered the “indirect effect” and that mediated by all other factors constituted the “direct effect”. The statistical significance of the indirect effects was assessed by the post-estimation “medsem” command[1] in STATA which utilizes the Zhao, Lynch & Chen's approach[2] to testing mediation through the Monte-carlo simulations (5000 reps).

Among patients with pre-existing ASCVD, we measured the association of statin use, classified according to the intention-to-treat and per-protocol definitions, and all-cause mortality and infection-related mortality using separate univariable and multivariable Cox regression models. In order to appropriately assign statin exposure, only patients who survived beyond the first month post-TB treatment initiation were included. Sensitivity analyses were conducted by: i) including patients who died within the first month of TB treatment; and ii) excluding patients who did not receive the required dose of statin from the comparison group. Statistical analyses were performed using STATA/IC 16.0 software (StataCorp, College Station, Texas).

References

1. Mehmetoglu M. Medsem: A Stata package for statistical mediation analysis. *Int J Comput Econ Econom*. 2018;8(1):63-78. doi:10.1504/IJCEE.2018.088321
2. Zhao X, Lynch JG, Chen Q. Reconsidering Baron and Kenny: Myths and Truths about Mediation Analysis. *J Consum Res*. 2010;37(2):197-206. doi:10.1086/651257

Supplementary table 1: Association of statin use with mortality in patients with ASCVD after excluding patients who died in the first month of TB treatment

Characteristic	Statin Use (Intention-to-treat)						Statin Use (Per-protocol)					
	Unadjusted HR	(95%CI)	p-value	Adjusted HR#	95%CI	p-value	Unadjusted HR	(95%CI)	p-value	Adjusted HR#	95%CI	p-value
All-cause mortality	0.46	0.23-1.01	**0.054**	0.41	0.17-0.99	**0.049**	**0.70**	**0.33-1.47**	**0.350**	**0.62**	**0.26-1.50**	**0.291**
Infection-related mortality	0.38	0.12-1.25	**0.111**	0.38	0.11-1.36	**0.136**	**0.55**	**0.17-1.81**	**0.325**	**0.58**	**0.16-2.07**	**0.403**

Adjusted for age, gender, body mass index (BMI), diabetes mellitus (DM), hypertension (HTN), cancer, chronic kidney disease (stage 3-5), asthma, chronic obstructive pulmonary disease (COPD), liver cirrhosis, transplant status, baseline sputum AFB status, cavitary disease at baseline, metformin use, and calcium channel blocker use.

Supplementary table 2: Association of statin use with mortality in patients with ASCVD after excluding patients who did not receive the required dose of statin from the comparison group.

Characteristic	Statin Use (Intention-to-treat)						Statin Use (Per-protocol)					
	Unadjusted HR	(95%CI)	p-value	Adjusted HR#	95%CI	p-value	Unadjusted HR	(95%CI)	p-value	Adjusted HR#	95%CI	p-value
All-cause mortality	0.42	0.29-0.92	0.026	0.40	0.19-0.84	0.015	0.64	0.35-1.17	0.147	0.49	0.23-1.04	0.063
Infection-related mortality	0.56	0.27-1.18	0.126	0.44	0.18-1.08	0.074	0.66	0.29-1.44	0.293	0.49	0.19-1.31	0.156

Adjusted for age, gender, body mass index (BMI), diabetes mellitus (DM), hypertension (HTN), cancer, chronic kidney disease (stage 3-5), asthma, chronic obstructive pulmonary disease (COPD), liver cirrhosis, transplant status, baseline sputum AFB status, cavitary disease at baseline, metformin use, and calcium channel blocker use.
